# Acute Mountain Sickness Is Associated With a High Ratio of Endogenous Testosterone to Estradiol After High-Altitude Exposure at 3,700 m in Young Chinese Men

**DOI:** 10.3389/fphys.2018.01949

**Published:** 2019-01-25

**Authors:** Xiao-Han Ding, Yanchun Wang, Bin Cui, Jun Qin, Ji-Hang Zhang, Rong-Sheng Rao, Shi-Yong Yu, Xiao-Hui Zhao, Lan Huang

**Affiliations:** ^1^Department of Health Care and Geriatrics, Lanzhou General Hospital of Lanzhou Military Region, Lanzhou, China; ^2^Department of Geriatric Cardiology, Chinese PLA General Hospital, Beijing, China; ^3^Department of Cardiology, Xinqiao Hospital, Army Medical University (Third Military Medical University), Chongqing, China; ^4^Department of Ultrasonography, Xinqiao Hospital, Army Medical University (Third Military Medical University), Chongqing, China

**Keywords:** testosterone, estradiol, acute mountain sickness, erythropoiesis, high-altitude exposure

## Abstract

**Background:** A large proportion of populations suffer from acute mountain sickness (AMS) after exposure at high altitude. AMS is closely related with age and gender implying that the sex hormones may play critical roles in AMS. Our observational study aimed to identify the association between the endogenous testosterone (T), estradiol (E2) and AMS.

**Methods:** A total of 113 subjects were recruited in 2012. The participants were evaluated at 500 m and after acute (1 day) and short-term (7 days) high-altitude exposure at 3,700 m. The subjects also completed a case report form questionnaire and underwent blood pressure measurements and an echocardiography examination. The red blood cell (RBC) count, Hb concentration ([Hb]), hematocrit (HCT), E2, T, and erythropoietin (EPO) were measured.

**Results:** Upon acute high-altitude exposure, E2 and EPO were significantly lower in AMS^+^ group, and T/E2 and stroke volume were higher. On the 1st day, AMS score correlated positively with the T/E2 ratio while it negatively correlated with E2. After 7 days at 3,700 m, the AMS^+^ subjects had higher erythropoietic parameters: EPO, T, and T/E2 were significantly higher in the AMS^+^ group. [Hb], RBC count, HCT, EPO, T and T/E2 were also correlated with AMS score. EPO, HCT, and the RBC count were also correlated with T/E2. Regression analyses indicated that T/E2 significantly correlated to AMS score and T/E2 on the 1st day was an independent predictor for AMS on the 7th day.

**Conclusion:** AMS was correlated with T/E2 ratio and EPO. After short-term exposure, higher T/E2 may contribute to AMS together with EPO *via* erythropoiesis. Furthermore, T/E2 level at high altitude in the early stage was an independent predictor for AMS in the latter stage.

## Introduction

Acute mountain sickness (AMS) has been defined as a syndrome involving headache, dizziness, gastrointestinal symptoms, insomnia and fatigue after arrival at high-altitude (>2500 m) (Imray et al., 2010; Bartsch and Swenson, 2013). The underlying mechanisms of AMS, which may involve the ventilation response, circulatory alteration and disequilibrium in the endocrine system induced by hypobaric hypoxia, have not been fully elucidated (Hackett and Roach, 2001; Imray et al., 2010; Davis et al., 2011; Bartsch and Swenson, 2013). The respiratory and cardiovascular aspects of AMS have been studied for many years; however, attention should be given to the endocrine aspects, particularly the effects of sex hormones and hematopoiesis on AMS (Naeije, 2010; Oliver et al., 2012). The endocrine system has been proposed to be of critical importance for the high-altitude adaptation process, which involves regulation of the hemoglobin (Hb) level (Gonzales, 2011). Previous studies have identified that several genes are associated with high-altitude adaptation and three of them are associated with [Hb] indicating that high-altitude adaptation and diseases may be associated with hematopoiesis and genetic factors (Simonson et al., 2010; Lorenzo et al., 2014). Testosterone (T) and E2 are the two most active sex hormones and are suggested to participate in AMS, based on the gender differences in the incidence of AMS (Harris et al., 1966; Wu et al., 2012).

Erythropoiesis is a hormonally regulated process that involves at least two hormones, EPO and testosterone (Richmond et al., 2005; Bachman et al., 2013). The crucial regulator of erythrocyte production, EPO, is elevated after short-term high-altitude exposure and hypoxia treatment. However, EPO does not increase proportionally with exposure time, and it even shows a tendency to decrease after reaching a peak (Koury, 2005; Gunga et al., 2007). Testosterone also enhances hemopoiesis and participates in many physiological functions, including erythropoiesis (Congote et al., 1977; Coviello et al., 2008; Rochira et al., 2009; Gonzales et al., 2011; Oskui et al., 2013; Shin et al., 2016). Though it has been suggested that high T concentrations, could compromise adaptation to high altitude, an association between T and EE in CMS has also been demonstrated (Gonzales et al., 2009, 2011), which indicates that T may participate in mountain sickness through its effects on erythropoiesis. The possibility that the variation in the normal physiological range of T concentration modulates men’s adaptation to hypobaric high-altitude hypoxia by stimulating Hb production and/or causing respiratory disturbances and exacerbating hypoxemia has been suggested (Gonzales, 2011, 2013; Gonzales et al., 2011b). E2 exhibits opposite effects that limit EPO and RBC production, and testosterone is converted to E2 by aromatase directly or after exerting its action. Both pathways of the conversion will reduce T biological actions. Thus, the T/E2 ratio may be used as a indicator of sex hormones balance in many diseases.

Numerous investigations have been performed on the acute responses in respiratory and cardiovascular systems and on EE in CMS (Ou et al., 1994; Cornolo et al., 2004; Iturriaga et al., 2005; Imray et al., 2010); however, fewer studies have given proper attention to erythropoiesis, which is balanced by sex hormones and EPO upon acute high-altitude exposure, and their roles in AMS. We postulate that the indicator of the sex hormone balance, ratio of T to E2, may participate the pathophysiological process of AMS. Furthermore, T/E2 represent the balance of T and E2 which may indicate diseases. The current study aimed to identify the relationship between endogenous T, E2, T/E2, and AMS upon acute and a short-term of high-altitude exposures.

## Materials and Methods

### Participants and Procedures

#### Participants

Altogether, 113 subjects (6 were lost to follow-up) participated in the study according to the inclusion and exclusion criteria. The inclusion criteria were that the subjects be healthy males who were between 18 and 60 years old. The exclusion criteria were as follows: respiratory diseases, cardiovascular diseases, neuropsychosis, cerebrovascular diseases, malignant tumor, diseases of the liver, and diseases of the kidneys.

Subjects who participated in the study voluntarily were fully familiar with the purpose and procedures of this study and signed informed consent before the field trials. The study was reviewed and approved by the Ethics Committee of Xinqiao Hospital, the Third Military Medical University. The trials have been performed in accordance with the ethical standards laid down in an appropriate version of the Declaration of Helsinki.

#### Procedures

The participants were recruited in June 2012. The field trials were performed in June 2012 within 18–24 h after the participants arrived at 3,700 m by plane, which occurred within a 2-h window, and on the 7th day after their arrival. The baseline data were recorded at 500 m within 1 week prior to departure for the high-altitude trials.

Structured CRF questionnaires were used to record demographic data (age, BMI, smoking and alcohol consumption) and the symptoms of AMS including headache (0 = no headache; 1 = mild headache; 2 = moderate headache; 3 = severe headache), dizziness (0 = no dizziness; 1 = mild dizziness; 2 = moderate dizziness; 3 = severe dizziness), gastrointestinal symptoms (0 = without and 1 = with any gastrointestinal symptom), insomnia (0 = normal sleep pattern; 1 = worse than usual; 2 = wake up multiple times during the night and 3 = extreme difficulty sleeping) and fatigue (0 = without fatigue and 1 = with fatigue). AMS was diagnosed by the LLS. Subjects were diagnosed with AMS if they had a headache upon arriving at high altitude and recorded an LLS score > 3. The SpO_2_ and HR were measured by a pulse oximeter (NONIN-9550, Nonin Onyx, United States). The SV, EF, and CO were measured by ultrasonography using the S5-1 cardiac probe (CX50, Philips, United States). CIn were calculated using CO divided by body surface area.

The venous blood samples were obtained from all participants between 8 and 10 am after an overnight fast (at least 12 h). The measurements of RBC count, [Hb], HCT, PLT, PCT, E2, T, and EPO were as follow: Blood samples were obtained after a rest of 30 min by vein puncture and collected in tubes containing EDTA. Routine blood tests (RBC count, [Hb] and HCT) were performed using an automated hematology corpuscle analyzer (AU400; Olympus Optical, Co., Tokyo, Japan) at 3,700 m in the Laboratory Department in Army Brigade Hospital immediately after the blood samples were obtained. Routine blood tests [RBC count, [Hb] and HCT] were performed using an automated hematology corpuscle analyzer (AU400; Olympus Optical, Co., Tokyo, Japan). The venous blood samples were allowed to clot for 30–60 min and were then centrifuged at 1,000 *g* for 10 min at room temperature to obtain serum, which was immediately stored at -20°C until the assays for hormonal analysis. The E2, T, and EPO were measured in duplicate using commercially available ELISA kits (human E2, T and EPO kits, BlueGene Ltd., China). To measure the exact concentrations of E2, T, and EPO, each sample was assayed in duplicate. All of the biochemical variables (except blood routine examinations) were measured from the blood specimens in the Clinical Laboratory Department, Xinqiao Hospital, Army Medical University, following the criteria of the World Health Organization Lipid Reference Laboratories.

### Statistical Analysis

**FIGURE 1:**
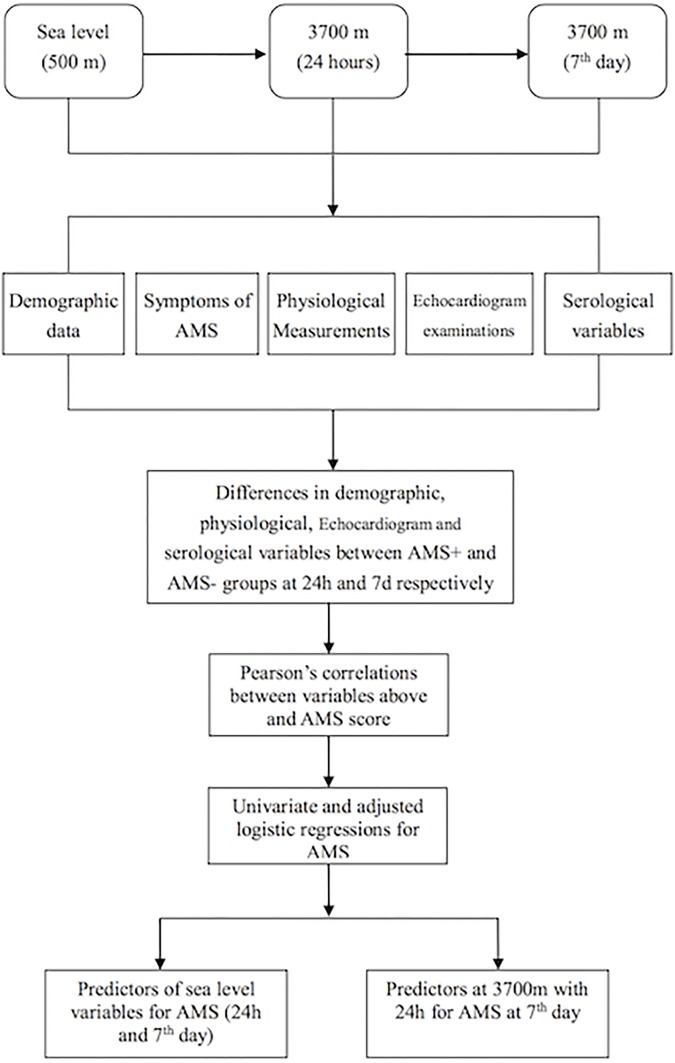
The flow chart of this study.

The CRFs were excluded if the required items were not completed. The mean concentrations of E2, T, and EPO were calculated from duplicate wells in the ELISA plates. Because all variables (age, BMI, [Hb], RBC counts, HCT, E2, T, EPO, T/E2, HR, SV, EF, CO, and CIn) were normally distributed, they are expressed as mean ± standard deviation (SD). The changes in [Hb], RBC count, HCT, E2, T, EPO, T/E2, HR, SV, EF, CO, and CIn from sea level to 3,700 m (acute and short-term exposures) were evaluated by analyses of variance for repeated measurements. The variables mentioned above were compared between the AMS^+^ and AMS^-^ groups by the independent-samples Student’s *t*-test on the 1st and 7th days at 3,700 m, respectively. The enumerated data (smokers and AMS) are expressed as the rate of occurrence (%). Additionally, the relationships between the AMS score and the above-mentioned parameters at 3,700 m were obtained by Spearman’s correlation analyses. Multiple regression analyses were also performed after the AMS scores were normalized by natural logarithm transformation. Variable with *p* less than 0.3 was selected for further adjusted analyses after each of them was analyzed by univariate Logistic regression. The statistical analyses were performed using SPSS 19.0 software for Windows. *P* ≤ 0.05 was considered statistically significant. The detailed statistical methods and flow chart are summarized in Figure 1.

## Results

The age of the subjects in this study was 23.08 ± 4.17 years, and the BMI was 21.56 ± 2.19 kg/m^2^. Overall, the incidences of AMS upon acute exposure and upon 1-week exposure at 3,700 m were 57.9%b (62/107) and 19.6% (21/107), respectively.

**FIGURE 2:**
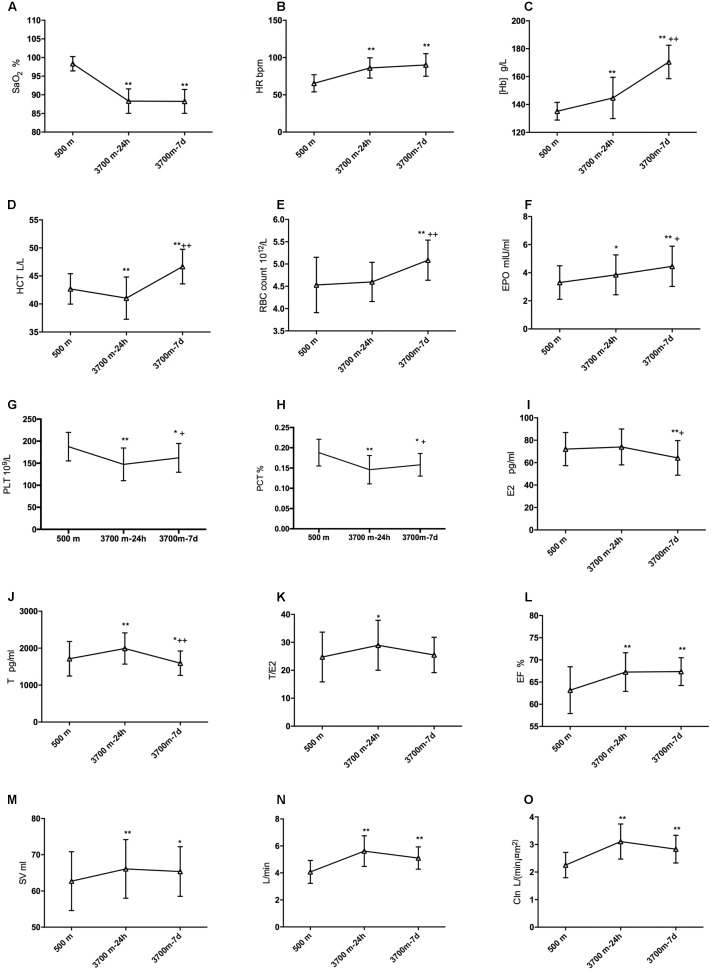
Modifications of sex hormones, EPO and hematopoiesis after high-altitude exposure. Compared with baseline: ^∗^*p* is 0.05 or less; ^∗∗^*p* is 0.01 or less. Compared with 24-h level: ^+^*p* is 0.05 or less; ^++^*p* is 0.01 or less. **(A)** Modification of SpO_2_; **(B)** Change of HR; **(C)** Modification of [Hb]; **(D)** Change of HCT; **(E)** Change of EBC count; **(F)** Change of EPO; **(G)** Change of E2; **(H)** Change of T; **(I)** Change of T/E2; **(J)** Change of EF; **(K)** Change of SV; **(L)** Change of CIn; **(M)** Change of SV after high altitude exposure; **(N)** Change of CO after high altitude exposure; and **(O)** Change of CIn after high altitude exposure.

The alterations of SpO_2_, HR, [Hb], RBC count and HCT after exposures to 3,700 m were revealed in Figures 2A–E. The erythrocytosis-regulated hormone EPO increased significantly after acute exposure (3.31 ± 1.20 and 3.85 ± 1.42 mIU/ml, *p* = 0.045) and increased even further after short-term exposure to 3,700 m (4.46 ± 1.44 mIU/ml, Figure 2F). Furthermore, the PLT and PCT were decreased on the 1st day and recovered on the 7th day (Figures 2G,H). E2 had a slight increase (72.11 ± 14.78 vs. 74.07 ± 16.00 pg/ml, but not significantly) and T (1713.16 ± 468.06 vs. 1992.29 ± 423.96 pg/ml) had a significant increase from baseline to the acute time point. Both fell to a lower level than baseline at 7 days (64.26 ± 15.41 and 1592.82 ± 330.55 pg/ml, respectively) (Figures 2I,J). Though T decreased after a sharp elevation and E decreased after 7 days, the ratio of T to E2 increased significantly (24.76 ± 8.91 vs. 28.96 ± 8.93) from baseline to 24 h, and it remained at a relatively high level on the 7th day (25.48 ± 6.34) (Figure 2K). We also found that EF and SV increased after arrival at 3,700 m remained significantly increased after 7 days (Figures 2L,M). Both CO and CIn increased at 24 h and remained significantly increased after 7 days (Figures 2N,O).

Upon acute high-altitude exposure, E2 and EPOwere significantly lower in the AMS^+^ group than in the AMS^-^ group, while the T/E2 ratio was higher in the AMS^+^ group without significant changes in T. SV was significantly lower in the AMS^+^ group than the AMS^-^ group. However, SpO_2_, HR, CO, [Hb], RBC count and HCT showed no significant differences between AMS^+^ and AMS^-^ individuals, as indicated in Table 1.

**Table 1:** Differences of sex hormones, EPO, cardiovascular, hematopoietic and erythropoiesis between AMS^+^ and AMS^-^ groups.

	3,700 m-24 h			3,700 m -7 days		
	AMS^-^ (45)	AMS^+^ (62)	*p*	AMS^-^ (86)	AMS^+^ (21)	*p*
SpO_2_	88.77 ± 2.80	87.98 ± 359	0.227	87.93 ± 2.50	88.50 ± 3.90	0.481
[Hb]	145.73 ± 15.43	144.00 ± 14.58	0.553	169.24 ± 12.29	175.71 ± 9.32	0.026
RBC	4.62 ± 0.40	4.59 ± 0.48	0.659	5.04 ± 0.45	5.56 ± 0.46	0.025
HCT	41.34 ± 3.85	40.84 ± 3.75	0.663	46.29 ± 3.12	48.27 ± 2.38	0.008
E2	78.26 ± 14.94	71.02 ± 16.17	0.020	62.65 ± 15.49	63.32 ± 15.10	0.872
T	1723.59 ± 557.55	170.59 ± 395.37	0.854	1537.00 ± 301.200	1821.45 ± 353.88	<0.001
T/E2	25.55 ± 13.01	31.32 ± 15.80	0.045	25.32 ± 6.45	29.49 ± 6.45	0.009
EPO	4.08 ± 1.63	3.68 ± 1.28	0.039	4.29 ± 1.38	5.12 ± 1.50	0.017
HR	84.66 ± 13.08	88.41 ± 13.42	0.156	90.74 ± 15.72	87.93 ± 11.09	0.444
EF	67.11 ± 3.57	67.37 ± 4.86	0.762	67.10 ± 3.19	68.48 ± 2.60	0.071
SV	68.78 ± 7.17	64.16 ± 7.94	0.002	64.91 ± 6.74	67.19 ± 7.13	0.172
CIn	3.06 ± 0.63	3.14 ± 0.65	0.646	2.80 ± 0.51	2.94 ± 0.46	0.266
CO	5.56 ± 1.15	5.66 ± 1.13	0.530	5.03 ± 0.82	5.40 ± 0.80	0.067


**SpO_*2*_, %; HR, beat per min; E2, pg/ml; EPO, mIU/ml; T, pg/ml; [Hb], g/L; RBC, 10^*12*^/L; HCT, l/L; SV, ml; EF, %; CO, L/min and CIn, L/(min ⋅ m^*2*^); PCT, l/L; PLT, 10^*9*^/L*.*

After short-term exposure to 3,700 m, the AMS^+^ group had higher erythrocytosis parameters. Specifically, the AMS^+^ subjects showed significantly higher [Hb], RBC count and HCT (all *p*-values < 0.05). We also observed that T and T/E2 were higher in AMS^+^ individuals than in AMS^-^ individuals, whereas E2 showed no differences between the two groups. In contrast to the data obtained after 24 h at high altitude, EPO was higher in the AMS^+^ group than in the AMS^-^ group after 7 days. The hemodynamic parameters showed no differences between the AMS^+^ and AMS^-^ groups (all *p*-values > 0.05, Table 1).

After a 24 h-exposure to 3,700 m, both T (*p* < 0.05) and T/E2 (*p* < 0.05) were highly correlated with the AMS score, and E2 (*p* < 0.05) was negatively correlated with the AMS score (Table 2). Additionally, the AMS score on the 1st day at 3,700 m was correlated with SV (*p* < 0.05) and HR (*p* < 0.05).

**Table 2:** Relationship between AMS score and sex hormones, EPO, echocardiography and erythropoiesis at 3,700 m.

	SpO_2_	T/E2	[Hb]	RBC	HCT	E2	EPO	T	HR	EF	SV	CO	CIn
**3,700 m-24 h**													
*r*	–0.122	0.273	–0.036	–0.082	–0.038	–0.311	–0.110	0.029	0.289	–0.002	–0.304	0.083	0.061
*p*	0.215	0.005	0.713	0.403	0.695	0.001	0.258	0.767	0.003	0.978	0.001	0.396	0.533
**3,700 m-7 days**													
*r*	0.030	0.221	0.201	0.257	0.187	–0.009	0.201	0.171	–0.015	0.091	0.021	0.050	–0.025
*p*	0.760	0.015	0.030	0.004	0.054	0.905	0.038	0.077	0.877	0.351	0.734	0.613	0.799


*T/E2 was the only variable significantly related to AMS score after 24 h and 7 days high-altitude exposure.*

On the 7th day at 3,700 m, the AMS score was closely correlated with [Hb] (*r* = 0.206, *p* = 0.033), RBC (*r* = 0.307, *p* = 0.001) and HCT (*r* = 0.275, *p* = 0.004). Furthermore, T/E2 had a positive relationship with the AMS score (*r* = 0.241, *p* = 0.012). More importantly, the AMS score was correlated with EPO (*r* = 0.210, *p* = 0.030) (Table 2).

Among the erythropoietic and hemodynamic parameters, SV was significantly correlated with EPO (*r* = 0.199, *p* = 0.039) and T/E2 (*r* = -0.236, *p* = 0.015) on the 1st day at 3,700 m. The RBC count had significant positive relationships with EPO (*r* = 0.293) and T/E2 (*r* = 0.281, *p* = 0.003) after 7 days of exposure to high altitude. A significant relationship between EPO and T/E2 (*r* = 0.193, *p* = 0.046) was also observed (Table 3).

**Table 3:** Associations among erythropoiesis, EPO, echocardiography, and sex hormones.

	EPO		E2		T		T/E2	
	*r*	*p*	*r*	*p*	*r*	*p*	*r*	*p*
**3,700 m-24 h**							
EPO	/	/	–0.171	0.078	–0.044	0.652	0.162	0.099
Hb	–0.005	0.960	0.044	0.651	0.170	0.079	–0.007	0.940
RBC	–0.006	0.955	0.039	0.687	0.085	0.385	0.045	0.646
HCT	–0.018	0.856	0.043	0.662	0.093	0.342	0.052	0.598
EF	–0.132	0.174	–0.094	0.335	–0.071	0.468	0.096	0.330
SV	0.199	0.039	0.061	0.533	–0.037	0.707	–0.236	0.015
CO	0.029	0.765	–0.005	0.958	–0.010	0.916	–0.072	0.465
CIn	0.026	0.789	–0.052	0.596	–0.036	0.713	–0.070	0.476
**3,700 m-7 days**							
EPO	/	/	–0.026	0.789	0.148	0.129	0.193	0.046
Hb	0.105	0.281	–0.097	0.323	0.087	0.372	0.161	0.098
RBC	0.293	0.002	–0.146	0.133	0.164	0.092	0.281	0.003
HCT	0.099	0.309	–0.104	0.287	0.127	0.194	0.203	0.036
EF	0.023	0.810	0.084	0.390	0.098	0.317	–0.068	0.485
SV	–0.057	0.557	0.045	0.644	0.092	0.346	0.026	0.792
CO	–0.017	0.862	0.034	0.731	0.138	0.155	<0.0001	0.999
CIn	–0.030	0.757	0.031	0.754	0.120	0.218	–0.018	0.855


Regression analyses demonstrated that EPO and T/E2 (*p* < 0.001) correlated with the AMS score after 24 h of exposure to 3,700 m, while HCT and T/E2 were related to the AMS after 7 days (Table 4). Univariate regressions showed that E2 and SV at sea level were associated with AMS on the 1st day whereas SpO_2_, RBC and [Hb] can be included in the adjusted regression to identify the predictor for AMS on the 7th day (Table 5). Regarding to variables within 24 h at 3,700 m, only SV and T (T/E2) entered adjusted Logistic analyses. The adjusted Logistic regressions showed that baseline RBC count can predict AMS on the 7th day. Furthermore, T/E2 on the 1st day at 3,700 m is an independent predictor for AMS after 7 days’ exposure (Table 6).

**Table 4:** Multiple linear regressions at 3,700 m.

AMS score#	β	*t*	*p*-value	95%CI lower bound	95%CI upper bound
**3,700 m-24 h**					
T/E2	0.044	3.131	0.003	0.017	0.076
SV	–0.075	–2.525	0.008	–0.131	–0.021
EPO	–0.345	–2.152	0.022	–0.684	–0.054
**3,700 m-7 days**					
T/E2	0.063	2.040	0.047	0.004	0.127
HCT	0.158	2.476	0.018	0.031	0.285


*^#^Normalized by natural logarithm transformation.*

*95%CI lower bound and 95%CI upper bound are the upper and lower limits of the 95% confidence interval.*

*β, regression coefficient.*

*t, statistic of T-test for the regression.*

**Table 5:** Univariate Logistic regression for AMS.

	AMS (3,700 m-24 h)					AMS (3,700 m-7 days)				
	β	OR	95%CI		*p*	β	OR	95%CI		*p*
**Variable at sea level**					
SBP	0.017	1.016	0.983	1.054	0.299	–0.007	0.991	0.876	1.018	0.765
DBP	0.004	1.004	0.966	1.043	0.834	–0.015	0.980	0.940	1.022	0.375
HR	0.002	1.002	0.966	1.040	0.982	–0.006	0.994	0.956	1.034	0.788
SpO_2_	0.073	1.077	0.690	1.681	0.831	–0.259	0.772	0.482	1.236	0.281
E2	0.003	1.003	0.998	1.009	0.250	0.001	1.001	0.995	1.007	0.767
EPO	–0.001	0.999	0.933	1.068	0.968	–0.070	0.932	0.866	1.004	0.063
T	0.010	1.010	0.851	1.199	0.912	0.097	1.102	0.908	1.336	0.325
T/E2	–0.010	0.990	0.947	1.036	0.670	0.019	1.020	0.972	1.070	0.430
RBC	0.127	1.136	0.592	2.179	0.701	0.918	2.503	1.157	5.414	0.020
HB	0.015	1.016	0.955	1.081	0.619	–0.052	0.949	0.890	1.012	0.112
HCT	0.034	1.039	0.897	1.204	0.606	–0.065	0.938	0.801	1.097	0.420
EF	0.010	1.011	0.937	1.091	0.772	0.029	1.029	0.946	1.119	0.503
SV	0.044	1.051	0.993	1.112	0.088	–0.163	0.849	0.503	1.436	0.542
**Variable at 3,700 m (24 h)**					
SBP	0.015	1.016	0.977	1.055	0.429	0.008	1.008	0.969	1.049	0.697
DBP	0.017	1.017	0.975	1.060	0.434	0.018	1.018	0.974	1.064	0.419
HR	0.023	1.024	0.991	1.057	0.156	0.004	1.004	0.972	1.037	0.809
SpO_2_	–0.101	0.904	0.791	1.034	0.142	–0.030	0.970	0.851	1.105	0.647
E2	–0.006	0.994	0.989	1.000	0.035	0.000	1.000	0.995	1.005	0.996
EPO	–0.064	0.938	0.882	0.997	0.039	–0.016	0.984	0.925	1.046	0.606
T	0.089	1.093	0.963	1.242	0.169	0.119	1.126	0.999	1.269	0.052
T/E2	0.037	1.038	0.999	1.078	0.053	0.024	1.024	0.996	1.054	0.090
RBC	–0.001	0.999	0.399	2.501	0.998	–0.297	0.743	0.280	1.968	0.550
HB	–0.007	0.993	0.965	1.021	0.609	0.004	1.004	0.975	1.034	0.805
HCT	–0.023	0.977	0.877	1.088	0.670	0.024	1.024	0.912	1.151	0.685
EF	0.043	1.044	0.950	1.148	0.373	–0.024	0.976	0.882	1.079	0.636
SV	–0.091	0.913	0.861	0.968	0.002	–0.043	0.958	0.907	1.012	0.126


*The variable with p-value less than 0.3 was selected for the adjusted Logistic regression.*

*β, regression coefficient.*

*OR, odds ratio.*

**Table 6:** Predictive roles of sex hormones in AMS (adjusted regression).

	β	OR	95%CI		*p*
For AMS (7 days)					
RBC	0.840	2.030	1.005	5.012	0.033
**Variables at 3,700 m (24 h)**					
For AMS (7 days)					
T/E2	0.128	1.124	1.004	1.23	0.045


*RBC at sea level predicts AMS independently while T/E2 at 3,700 m on the 1st day is the independent predictor for AMS after 7 days.*

## Discussion

We have found that the AMS was closely associated with T/E2 and the T/E2 may predict AMS in a short term of time in this study.

### Associations Between AMS and Sex Hormones, EPO, and Hematopoiesis

Few studies have examined the relationships among sex hormones, EPO, hematopoiesis, and AMS. Most reports have demonstrated that CMS is characterized by high T concentrations and erythropoiesis, mainly due to the relationships among the T level, erythropoiesis and the symptoms of headache and sleep disturbance (Ou et al., 1994; Gonzales et al., 2009, 2011; Gonzales, 2011, 2013; Ekart et al., 2013). This evidence suggests that T and erythropoiesis may also participate in the pathogenesis of AMS. Hence, we observed that AMS was characterized by higher T, EPO, HCT, RBC count and [Hb] after 7 days at high altitude, though the T, HCT, RBC count and [Hb] were not significantly different between the AMS^+^ and AMS^-^ groups following acute exposure.

These results partly agree with the previous findings that high serum T and Hb are adequate for acclimatization due to the improvement of oxygen transport, while even higher levels of T and Hb are associated with EE (Gonzales, 2011; Ekart et al., 2013), which may account for the positive relationships between T/E2 ratio and AMS score on the 1st day as well as association of after 7 days of exposure. However, E2 was higher in the AMS^-^ group than in the AMS^+^ group, which may suggest that E2 may play a protective role in AMS upon acute exposure. EPO was also higher in the AMS^-^ group upon acute exposure, which suggests that in the acute phase, higher E2 and EPO may be the primary protective factors against AMS, perhaps due to the respiratory action of E2 and the non-hematopoietic effect of EPO (see below). Furthermore, E2 may protect individuals from AMS *via* the relief of pulmonary artery contraction through its depressive effect on pulmonary endothelin-1 induced by hypoxia (Earley and Resta, 2002). Though both EPO and E2 appear to protect against AMS, E2 downregulates HIF response genes, including *EPO* (Earley and Resta, 2002). It is possible that the depression of EPO by E2 is attenuated by acute hypoxia. Thus, the preventive effect of E2 may also be ascribed to its indirect role in polycythemia and right ventricular functions in the acute phase.

High T and T/E2 are correlated with hypoventilation, sleep disturbances, EE and, in turn, the etiopathogenesis of CMS in natives (Gonzales et al., 2011b). E2 prevents CMS *via* its protective effects on cardiovascular and respiratory functions (Zabka et al., 2006; Mattingly et al., 2008). Thus, it is reasonable to postulate that T and E2 play opposing roles in AMS, including the hematopoietic and respiratory effects.

Though T/E2 at sea level cannot be used as independent predictors for AMS neither on the 1st day nor the 7th day, T/E2 values upon acute exposure at 3,700 m was shown to be an independent predictor for AMS after a short-time exposure.

### Sex Hormones May Participate in AMS via Hematopoietic or Non-hematopoietic Effect of EPO

The production of erythrocytes by erythropoiesis requires time, which is in accordance with our result that the RBC count was unchanged in the acute phase followed by a rise at 7 days. Previous study showed that the plasma volume and blood volume were decreased about 10–12% after acute high altitude exposure. However, they will be recovered in few days to 1 or 2 weeks at high altitude or after return to sea level. There was an interesting result that the HCT and PCT were decreased on the 1st day following by PCT remained significantly reduced after 7 days while HCT increased, which may be caused by the water-sodium retention in acute phase. This have been also reported in few studies which were not completely coincident with other researches. Also, in the days at high altitude, the HCT were significantly increased combined with the increased of [Hb] and RBC, may revealed that the decreases of plasma volume and blood volume have emerged. However, on the 7th day, the mean increase percentages of [Hb] and RBC were 24 and 12%, whilst the HCT evaluated only 9% compared with sea level. This mismatching of [Hb] or RBC with HCT may not be explained by the decrease of PV completely. Which may indicate the erythropoiesis process have started by EPO (peak level appeared on 2–4 days at high altitude). Indeed, it is important to measure the plasma volume and blood volume directly to evaluate the changes of them. However, we searched the published papers and note that the simplest and easily get methods as follow: PV = BV - RCV; BV = RCV^∗^100/Hct; RCV = tHb/MCHC^∗^100. In our study, it is difficult for us to measure the total tHb due the difficult of flied study at high altitude. Thus, we cannot directly evaluate PV in this study. Therefore, the following explanation of our results dependent on the increased amplitudes in [Hb], RBC count and HCT indirectly. That is, in the short terms of high altitude exposure, both of PV decrease and erythropoiesis have emerged. Further analysis showed that the erythropoiesis may be preponderant in our study, which needs further precise studies.

Acute mountain sickness was characterized by higher erythropoiesis after 7 days of exposure, but it was not associated with [Hb] or RBC count upon acute exposure to high altitude. What are the underlying mechanisms by which EPO participates in AMS during acute exposure, if not by promoting erythropoiesis? The non-hematopoietic effect of EPO has been revealed by studies that have reported its inotropic effect on the myocardium, which results in elevated EF (Riksen et al., 2008). This observation may be interpreted as a protective effect of EPO in the acute exposure phase, before erythropoiesis plays a major role, as indicated by the relationship between SV and EPO in the present study.

E2 *per se* has a protective effect on the cardiovascular system and a stimulatory effect on the respiratory system (Mattingly et al., 2008; Smith et al., 2014). Combined with the enhanced respiration effects of EPO, we can suggest that the regulation of the respiratory and cardiovascular systems by E2 and EPO may account for their roles in preventing AMS in the case of acute exposure.

However, after a short time, the modifications of RBC, HCT, and [Hb] induced by the synergism of T and EPO were of critical importance in AMS. The RBC count had a positive correlation with EPO, which was also positively correlated with T. T also has a direct erythropoiesis effect (Gonzales et al., 2009; Gonzales, 2011), which was revealed here by the positive associations of the RBC count and HCT with the T level. Furthermore, our novel observation that EPO was higher in the AMS^+^ group after 7 days of exposure but that it was lower in AMS^+^ individuals upon acute exposure may further indicate that EPO has a diphasic role in AMS, depending on the duration of hypoxic exposure. Thus, in the short-term condition, T on its own and in combination with EPO induced erythropoiesis, perhaps enhancing the viscosity of the blood, which is harmful for acclimating to high-altitude hypoxia (Heinicke et al., 2003; Bachman et al., 2013; Ekart et al., 2013). Though previous studies indicate that EPO does not play a further important role in EE (Koury, 2005; Gunga et al., 2007), it may stimulate erythrocytosis when its effects are combined with the effects of T. T induces erythrocytosis via direct effects on bone marrow and indirect effects on EPO (Gonzales et al., 2009; Maggio et al., 2013), which finally results in the augmentation of [Hb], HCT, and RBC count that aggravate AMS.

**FIGURE 3:**
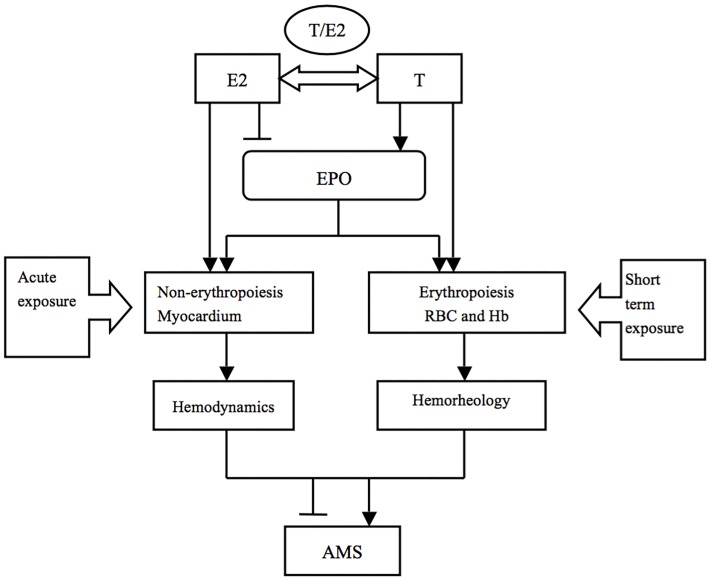
The potential mechanisms of sex hormones in AMS during different exposure durations. T and E2 may play various roles in AMS through different pathophysiological processes in a time dependent way.

We may conclude that E2 and EPO prevent AMS via the non-hematopoietic effect (inotropic effect on the myocardium, alterations in hemodynamics) in the acute phase, while T and EPO aggravated AMS through erythropoiesis (alterations in the hematological system) after short-term exposure (Figure 3). Additionally, E2 was still beneficial to individuals through the stimulation of ventilation in the latter phase.

### Disequilibrium of Endogenous E2 and T May Be Involved in AMS

T and E2 are the most active sex hormones in the human body. They play opposite roles in respiratory regulation and erythropoiesis (Ou et al., 1994). T is converted to E2 by aromatase after acting on erythrocytosis (Jiang et al., 2010). Thus, the ratio of T/E2, which is determined by the levels of T and E2 as well as aromatase, is more important than the absolute value of each sex hormone.

Though the effects of T/E2 on SpO_2_ have been reported, which our results do not corroborate the relationship between SpO_2_ and T/E2 (Gonzales and Villena, 2000) (which our results do not corroborate), as have the effects of T/E2 on respiratory regulation, erythrocytosis, and even CMS, few studies have focused on AMS. Our observations reveal that T/E2 was closely associated with the AMS score, which indicates the importance of T/E2 in the pathophysiological process of AMS. The erythropoietic effect of T occurred before aromatase could convert it into E2, which had no effect on erythropoiesis (even with a reverse action) (Zabka et al., 2006). On the other hand, T enhanced EPO production and erythropoiesis and induced hypoventilation, while E2 enhanced ventilation, increased SpO_2_ and inhibited EPO and RBC production. However, T may reduce these effects by down regulating the estradiol and progesterone receptors in response to ventilation, favoring hypoventilation and stimulating erythropoiesis (Ou et al., 1994; Zabka et al., 2006; Jiang et al., 2010).

Based on the evidence described above for CMS and our observations, it may be possible to regulate the T/E2 ratio by increasing the aromatization of T into E2, increasing aromatase expression or even applying E2 exogenously to prevent and treat AMS. Furthermore, T/E2 may participate in AMS *via* erythropoiesis, as indicated by the associations among T/E2, EPO, and RBC. The precise mechanisms by which T/E2 participates in AMS warrant further basic research.

### The Predictive Roles of T/E2 in AMS at High Altitude

The T/E2 at sea level was on an independent predictor for AMS on the 1st day or the 7th day at 3,700 m indicated by both univariate or adjusted regressions. However, the T/E2 on the 1st day at 3,700 m can predict AMS after 7 days indicating that the role of T/E2 was in progressing pathophysiological progress. Though, the sea level T/E2 failed to predict AMS, its association between AMS has been revealed in our present study. In addition, the predictive of T/E2 may be dependent on other risk factors that warrant further study.

## Conclusion

Acute mountain sickness was correlated with the ratio of T to E2 and the level of EPO. Our observations suggest that higher T/E2 may promote AMS by acting through or combining with EPO to affect erythropoiesis, however, the mechanisms by which this may occur warrant further study. Furthermore, though T/E2 at sea level was not an independent predictor for AMS, the T/E2 on the 1st day at 3,700 m was an independent predictor for AMS on the 7th day.

### Limitations

The subjects of this study were all young male workers, mostly aged between 16 and 40 years, which could perhaps generate a bias due to age or gender. The current study is an observational study with a small sample size, and larger sample sizes and mechanistic studies are needed.

## Author Contributions

LH and X-HD participated in the design of this research. X-HD, YW, and LH drafted the manuscript and performed the statistical analyses. LH, J-HZ, and JQ reviewed and revised this manuscript critically for important intellectual content. X-HD, J-HZ, BC, and X-HZ completed the collection of clinical data and performed the measurements of BP, HR, and the biomarkers (T, E2, and blood routine tests). R-SR and S-YY performed the echocardiography examinations.

## Conflict of Interest Statement

The authors declare that the research was conducted in the absence of any commercial or financial relationships that could be construed as a potential conflict of interest.

## Abbreviations

AMS^+^: with AMS
AMS^–^: without AMS
AMS: acute mountain sickness
BMI: body mass index
CIn: cardiac index
CMS: chronic mountain sickness
CO: cardiac output
CRF: case report form
DBP: diastolic blood pressure
E2: estradiol
EE: excessive erythrocytosis
EF: ejection fraction
EPO: erythropoietin
[Hb]: Hb concentration
HCT: hematocrit
HIF: hypoxia-inducible factor
HR: heart rate
LLS: Lake Louise self-assessment scoring system
PCT: plateletocrit
PLT: platelet count
RBC: red blood cell
SBP: systolic blood pressure
SpO_2_: pulse oxygen saturation
SV: stroke volume
T: testosterone
